# Nothing matters: the significance of the unidentifiable, the superficial and nonsense

**DOI:** 10.1080/17482631.2019.1684780

**Published:** 2019-11-01

**Authors:** Tore Dag Bøe, Inger Beate Larsen, Alain Topor

**Affiliations:** a Department of Psychosocial Health, University of Agder, Kristiansand, Norway; b Department of Social Work, Stockholm University, Stockholm, Sweden

**Keywords:** Mental health, recovery, qualitative research, hermeneutics, Ingold, Gumbrecht, Biesta

## Abstract

**Purpose:** The aim of this study is to explore the ways in which “small things” may be of importance for people with mental health difficulties.

**Method:** Empirical material from three different studies was reanalysed through a phenomenological, dialogical, approach.

**Results:** We discovered some paradoxical aspects of small things: i.e., they could be about “something” that was difficult or even *impossible to identify*. The unidentifiable could be about bodily, sensual experiences that are *superficial* (i.e., belonging to the surface). The interaction with others highlighted as significant could be about doing something fun, talking nonsense or kidding around, and hence not at all about making sense of something—a kind of important *nonsense*. We summarize these aspects in three themes: the importance of the *unidentifiable*, the *superficial* and *nonsense*. These aspects can be regarded as small things—even “nothings”—that make it possible “to stay in the world”.

**Conclusion:** We elaborate on the findings in relation to the following: Gumbrecht’s critique of the prevailing hermeneutic world-view with its idea that “interpretation is humankind’s exclusive way of relating to the world”, Ingold’s idea that social life is lived in relations of “interfacility” and hence a turn to surfaces is needed for a “restoration of social life”, and Biesta’s idea of existence as “coming into the world in the presence of others”.

“*We need to be alert whenever events shape themselves into narratives, for narratives belong to literature and not to life*” (Knausgaard, 2018, p. 534).

## Introduction

This study explores the ways in which “small things” may be of importance for people with mental health difficulties. To this end, interviews from three different empirical studies were reanalysed. In our discussions, after a first reading of the interviews, we described what caught our attention and found that there often seemed to be “something” that the participants were telling about that was not easily put into words but that had nevertheless made a strong impression and were referred to as being significant to them in their recovery struggles. We wanted to explore further whether this might be about something that in a sense was not possible to identify, something that escapes when being approached by the question “What is/was it?” If as researchers, we equal what is *real* with what can be *identified*, then we may become blind to important aspects in people’s lives. Important aspects may fall into a kind of nothingness. It could be that important things had happened in the lives of the interviewed people that were of a nature that evaded attempts at meaning-making and understanding. And if so, perhaps one could say “It’s something, but when trying to put it into words, *it’s nothing*”. Still, this “nothing” mattered much. Hence, our play on words in the title: “Nothing matters”.

### The hermeneutic world loss

Our exploration started with a search for small things that matter. As we became interested in the “nothings” outside what hermeneutics can capture, we turned to ideas found in Gumbrecht (2004), who argues that human sciences have ended up in a hermeneutic paradigm that stands in the way of including the bodily and material sides to human living. “Hermeneutic maximalists”, Gumbrecht (2004) says, “hold interpretation to be humankind’s exclusive way of relating to the world” (p. 55). His claim is that human living cannot be reduced to the ways in which we understand and give meaning to the world. Gumbrecht (2004) argues that this attention towards understanding, interpretation and meaning has led to “a loss of the world” (p. 49): i.e., the world and life that “meaning cannot convey” (p. 65).

Gumbrecht points out that communication materials have been bracketed in hermeneutic accounts, making such accounts blind to how communication takes place within the domain of materials and the body. Gumbrecht acknowledges that communication certainly produces *meaning* (i.e., an interpretive relation to the world), but suggests that communication also produces *presence* (i.e., a sensible, touchable relation to the world). A hermeneutic world-view preoccupied solely with meaning becomes blind to the materials of the world and how humans live their lives in a world of things and bodies in ongoing movement, perceivable and touchable at the surfaces. Our interest in the “nothings” that seemed to matter and Gumbrecht’s ideas provided a point of departure for this study. More specifically, we wanted to reanalyse interviews collected in earlier studies in a search for aspects we might have missed when having previously read the interviews through hermeneutic lenses.

### The quantitative fallacy … and a qualitative fallacy?

This approach may put us in an awkward position when it comes to seeing this study as a qualitative study. Is not the intent of qualitative research precisely to search for meaning and to understand, relying on the act of identifying and meaning-making? Is there a fallacy in the traditional qualitative approach?

The *quantitative fallacy* (also known as the *McNamara fallacy*) was formulated by the sociologist Yankelovich (1972) in response to the way McNamara, the US Secretary of Defence during the US war in Vietnam, believed that the US government could quantify their success in the Vietnam war through body counts. The US government ignored the reality and experiences of what happened in Vietnam because they blindly trusted their measures and models. More generally, Yankelovich points out a kind of blindness that follows a one-sided focus on what can be measured and quantified and describes the steps of such a process in the following way:

The first step is to measure whatever can easily be measured. This is OK as far as it goes. The second step is to disregard that which can’t be easily measured or to give it an arbitrary quantitative value. This is artificial and misleading. The third step is to presume that what can’t be measured easily really isn’t important. This is blindness. The fourth step is to say that what can’t be easily measured really doesn’t *exist*. This is suicide (Yankelovich, cited in O’Mahony, 2017, pp. 281–282).

One might say that this emphasizes the importance of a qualitative approach because qualitative research may recognize and articulate knowledge that falls outside the domain of quantities and measurement.

Our approach in this study is qualitative. Nevertheless, we would like to continue in line with Yankelovich’s argument and also question qualitative explorations. The argument, which we believe is in line with Gumbrecht’s critique of the hermeneutic world-view, could go like this: What is of importance and significance cannot be reduced to what can be identified, known and represented. Here follows an attempt to articulate this idea of a possible *qualitative fallacy* by rephrasing the original quantitative fallacy presented above:

The first step is to identify the events of life and put them into meaningful words. This is OK as far as it goes. The second step is to disregard that which cannot be identified and put into words in hermeneutical coherent ways. This is artificial and misleading. The third step is to presume that what cannot be identified and put into words really isn’t important. This is blindness. The fourth step is to say that what cannot be identified and put into words really doesn’t exist. This is suicide.

There may be crucial and significant aspects of living that fall outside what can be identified, understood and accounted for, not only in *quantitative* ways but also in *qualitative* ways. In this present study, we want to give attention to what may be omitted by the meaning-making enterprises of hermeneutically oriented qualitative approaches.

### “Nothing” in mental health research: findings outside hermeneutic and instrumental rationality

One could ask if qualitative research within the recovery tradition finds itself within a hermeneutic paradigm. In the sense that researchers ask for retrospect interpretations from those who are identified as recovered and ask for the narratives they create that elucidate the reasons or causes for their recovery. This could be characterized as a kind of rational instrumental hermeneutics. These retrospectively formulated experiences are interpretations asked for in interviews based on the idea that recovery is a goal and that various aspects can be identified as leading to this recovery. If the retrospective narratives are put to such a formula of cause and effect, qualitative recovery research ends up in the same instrumental rationality as experimental research; i.e., certain causes (instruments/methods) are believed to cause certain effects (outcomes).^1^

Most research in mental health and therapy operates within these prevailing rationalities. However, several studies highlight aspects that are outside both the instrumental and the hermeneutic rationalities in mental health.

In a review exploring *small things* in the recovery literature (Topor, Bøe, & Larsen, 2018), small things seemed to occur as important to a person’s well-being and development across many studies. These small things are not part of a treatment procedure, and are not meant to have an impact on the person’s problems or to contribute to their recovery process. They could be about seemingly casual and even invisible parts of a process, both in everyday life and in the interaction between the person and the professional. Topor and Denhov (2015) suggest the phrase “going beyond” and point to aspects such as how the collaboration is “emotionally charged” (p. 231), the experience that the professional “has seen something” in the user (p. 232) and experience “to be given value” (p. 233).

Based on fieldwork in the context of mental health care, Skatvedt and colleagues (Skatvedt, 2017; Skatvedt & Scheffels, 2012; Skatvedt & Schou, 2008) indicate the significance of various aspects outside of what they refer to as ‘ordinary therapeutic rationality’^2^: “messages beyond the spoken” (Skatvedt & Schou, 2008, p. 90), “breaches in the therapy frame” (Skatvedt & Schou, 2008, p. 95), “pauses as breaches and movement”, the significant togetherness found in “empty talk” (Skatvedt & Scheffels, 2012, p. 37), “empty gestures” (Skatvedt, 2017, p. 12), and “the beautiful in the commonplace” (Skatvedt & Schou, 2008, p. 88).

Several studies emphasize hope as a significant aspect (Biong & Ravndal, 2007; Herrestad & Biong, 2010; Sælør, Ness, Holgersen, & Davidson, 2014). Hope is a relation to something in the future, something “not yet” and in that sense, it is a “nothing”. Furthermore, hopelessness is closely related to mental health difficulties (Herrestad & Biong, 2010). A relation to what is not there, what is absent, as in the experience of having no future (Bøe et al., 2014).

Narrative approaches emerging from social constructionism suggest that the creation and recreation of narratives, meanings and understandings is the healing element in therapy (Larner, 2008). Perhaps this focus on co-creation of *meaning* misses something out? Within what is referred to as dialogical practices, increasing attention is being paid to aspects outside such hermeneutic meaning-making. Seikkula (2002, 2011) suggests that it is perhaps not the narratives created in the meetings that are of importance, but the *event* of narrating. The ways in which the interlocutors are given space to talk are as much a bodily, emotional and expressive event as a meaning-making event. Dialogue and communication should not be reduced to a matter of hermeneutics; dialogue is as much a matter of the ethics, expressivity and vitality of human living (Bøe et al., 2013, 2014, 2015; Rober, 2005; Seikkula & Trimble, 2005).

Studies within psychotherapy research suggest that relational aspects and common factors are more important than the method-specific ingredients (Norcross & Wampold, 2011; Wampold & Imel, 2015). We see new operationalizations of psychotherapy based on the identification and articulations of relational aspects and common factors. This new emerging relationship-based rationale in psychotherapy is questioned by Bertelsen and Bøe (2016) as they suggest that there may be “something” unoperationable that is “at play” in therapeutic encounters that escapes even this relationship-based view. They suggest that there may be a crucial uniqueness of the encounter that is about “the interruption of the work and the enterprises of the rational community” (p. 372). A “something” in the encounters that perhaps cannot be defined in positive ways (e.g., as identification of various relational components), but perhaps must be indicated negatively in terms of a break with the identifiable roles and norms of the therapeutic setting.

### Aim and research question

The starting point of this study was to explore the significance of what we refer to as *small things* in the lives of people with mental health problems. How is the significance of such small things described from a first-person perspective? Based on our interest in the aspects that seemed to matter much but were still difficult to put into words—or when words were found they seemed poorer and not able to grasp what mattered—we articulated the following research question: *What aspects of importance in the lives of people with mental health problems may fall outside a hermeneutic gaze?*

## Methods

### Speculative science

Ingold (2007, 2017a) suggests that science should be *speculative*. He proposed that science should not be reduced to describing, interpreting and contextualizing to *understand* people, but rather to engage in a study of the possibilities of life in the world together with people, to not merely produce knowledge, but to open us up to possibilities of life other than what we might have ever imagined (Ingold, 2007). This is also in line with the dialogical approach to qualitative analysis (Sullivan, 2012) used in the analysis of the interviews. From this, we could say that our present study has a speculative touch, offering ideas and concepts that have emerged from a mix of the voices in the interviews, theoretical ideas and our own ideas.

### Participants and data

The authors originally studied various aspects concerning mental health issues. Topor and colleagues studied recovery processes by interviewing people with severe mental health difficulties. In their study, 30 persons in recovery were interviewed in different parts of Sweden in a collaborative project between researchers and the main Swedish user movement (Schon, Denhov, & Topor, 2009). Larsen (2009) studied the impact that materiality in psychiatric institutions had on patients and staff. She conducted participant observation in five district psychiatric centres and interviewed 16 patients and 22 professionals. Bøe et al. (2014, 2015) studied processes of change related to mental health care for youths aged 16–18 years with no previous history of hospitalization receiving therapy from a hospital out-patient service. None of the studies registered formal diagnoses.

The data for this present study consisted of transcribed interviews from these three different research projects. Each of the three authors chose four interviews from our separate datasets and shared them for the purpose of a reanalysis in this new study. The interviews were selected based on each authors’ preliminary assessment of their relevance to our focus on the significance of small things in the described recovery processes. Thus, we obtained 12 interviews: eight with adults and four with adolescents.

### Ethical considerations

All three studies that provided data for this present study were conducted according to legal and ethical principles for research. Bøe and colleagues’ and Larsen’s studies were approved by the Norwegian Regional Committees for Medical and Health Research Ethics (refs S-03073 and 2973–2). Larsen’s study was also approved by the Norwegian Centre for Research Data (ref. 9925) Topor and colleagues’ study was approved by the Department of Social Work at the University of Stockholm. Participants in all studies gave their informed written consent to participate. In Bøe and colleagues’ study, the participants were 16–18 years old and their parents/guardians also gave their written consent. The interviews shared between the authors in this present study were all anonymized.

### Analyses

Analyses were conducted according to Sullivan’s (2012) phenomenological–dialogical approach. All three authors read through the interview material looking for descriptions of small things of significance for the recovery and well-being of the participants. We then met to discuss our impressions from this first reading. We made a preliminary discovery after this first reading, i.e., participants often seemed to be telling about something important, but this “something” of importance simultaneously seemed difficult to put into words. Phrases like “it was something about him” or “the way she spoke to me” or “just driving together” are examples of this difficulty. Furthermore, if words were found, we wondered if the found words had missed out on what was important and were poorer than the participants’ initially more hesitant and blurry articulations. We made this discovery early in the analytical process and it was used as an analytical lens in our further re-reading of the material.

We identified “key moments” throughout the material (Sullivan, 2012). This search for key moments was not solely based on the meaning content of utterances, but also included a mixture of what affected us as readers, and our sense of what seemed to matter most to the participants based on both their own implicit or explicit ways of pointing out the most important, and our assessment of the expressivity displayed when telling. Sullivan (2012) notes that exploring lived experiences should not be reduced to the *content* of what is said, but it should also include the *ways* things are said, the expressiveness and how what is said is laden with various feelings and vitality.

**Figure 1.**
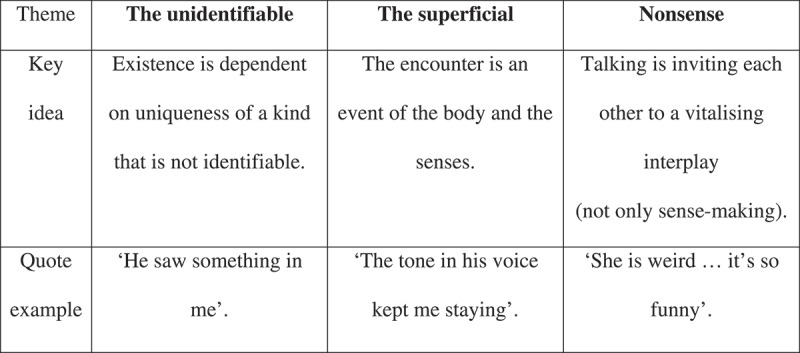
Nothing matters: aspects missing from the hermeneutic gaze.

In a next step, key moments were analysed by the first author, looking for possible themes. Preliminary themes were discussed with the other authors and through this dialogical process the findings were articulated in three themes: (1) the *unidentifiable*, (2) the *superficial* and (3) *nonsense*—that mattered (Figure 1). These concepts appeared to offer a way to highlight various aspects that seemed important, but could easily escape notice if analysed through a traditional hermeneutical gaze. For example, a boy pointed out a therapist as being very good but he seemed unable to give any elaboration on how the therapist was good other than saying that he was quirky. A girl told about how her friend was teasing her, and that this was important to her but could not give an account of why and how this was important.

## Findings: the unidentifiable, the superficial and nonsense that matters

### *“He saw something in me”*—the unidentifiable that matters

“He saw something in me” (T-1^3^), said one participant about a male staff member who meant a lot to him during a time when he was admitted to a psychiatric ward. What was this “something” that the staff member saw in the participant? When the interviewer followed the participant up about this, he tried to put it into words. What struck us was that it seemed difficult for him to find the right words and one might suspect that the words he suggested to explain what this “something” meant were poorer than his initial statement: “He saw something in me”.

Thus, small things could be about something—in an encounter, in a relationship, in an event or in a person—that may be “*outside*” *the identifiable*. First, in the sense that its significance is not about or dependent upon one understanding what it is, the significance is not about any (new) *understanding* of him/herself, others and the world around. Second, and more radically, what is referred to as “something” may even be about something outside, or prior to what can be identified and understood at all. We suggest this could be framed as *the unidentifiable that matters.*

The man said “I (…) walked away (…) I walked clinging to the walls … ? I didn’t say anything” (T-1). As if he wanted to be unnoticeable. He spoke about a “something” that the staff member Patrick^4^ saw *in him* as well as about a “something” *between them*, this man and himself.

There was *something* between Patrick and I. (…) He saw something in me that he liked. I stood my ground (…) [I]f he came too close I became horribly cruel and even growled. And Patrick liked that, he thought it was good because he saw, kind of, ‘aha’, there is something there after all. (T-1)

The man had in a sense withdrawn from the world into a kind of non-existence, even an attempt to merge with the surfaces of the surroundings. Literally, he was “clinging to the walls”. Nevertheless, Patrick approached him, wanted something from him and he got “something”: i.e., a growl. The man stood out. He stood forth as an event of existence in the etymological sense of the word (Latin existence, *ex* = forth and *sistere* = stand) (Online Etymology Dictionary, 2018). The “something” was as much about this uniqueness in itself coming into view in his response as a “something” in terms of a quality, competence or specific character of his personality. They did not leave each other in peace. Patrick defied the man’s attempt to be invisible and the man growled in defence. In this event, “something” that mattered to the man arose.

In a similar way, a boy (B-1) enthusiastically described the practitioner who helped him as “the world’s best psychologist”. Nevertheless, when he was asked what made this psychologist so good, he started out energetically as if it would be easy to explain, only to find out that it was not.

He is … he is like … sometimes I have said that he is … he is like … he is quirky actually [laughs loudly]. It’s hard to say how he is. … He is weird. He is a real … I would recommend him to anyone. … So, he is … he must be … No, he is simply the world’s greatest … psychologist. He is just insanely good. Well, it is … the way he … just to look …, just to look at him, you know [smiles and twists his body]. Just to see him, like, the way he looks. … He is … he is quirky, simply. … Unfortunately, I cannot describe him with any other words than that. (B-1)

There is this “something” that made this practitioner very important to the boy. But this quality seemed to escape the boy’s fumbling efforts to find the words. He ends up by saying that “he is quirky” and that you “just [have to] to see him, the way he looks”, indicating that it was a matter of being there and experiencing it, and not a matter of any identification of certain qualities, properties or characteristics of the practitioner.^5^

This unidentifiable “something” that participants seemed to want to bring forth as being important, but is difficult to describe seems to be about a kind of uniqueness. Maybe this is why one of the participants had to use proper names when talking about the staff of the ward where he is admitted: “Then there is Peter, I am a bit afraid of him sometimes. Solfrid, I don’t mind” (L-3).

If for example ‘Solfrid’ had been replaced by “staff member A” in the transcript, the uniqueness of “Solfrid” slips away or takes on another character. We may perhaps miss that Solfrid was singled out as unique in her existence and shift to thinking of her in terms of certain identifiable qualities of competence, actions, attitudes, values, and so on. A proper name presents particular challenges for theories of meaning because it answers the purpose of showing what thing or person that is talked about, but not telling anything else about the thing or person.

### *“It’s important to pat cows on their velvet muzzle”—*the superficial that matters

A man told about how his boss meant much to him.

I remember my boss …, he had this mild tone in his voice (…) those friendly eyes, that was what kept me staying. (T-2)

Interestingly, the participant did not seem to point to what his boss *did* for him, or to the *content* of what he said in their conversations. What his boss meant to him seemed to be based on another domain, i.e., of the body, senses, tone and gestures. In our reading of the interviews, we saw several descriptions of the significance of encounters with others, but not by pointing to what they said or what they did. What seemed important was *the way* they were spoken to, responded to or approached: i.e., the *tone* of voice and the *way* they looked at them.

Perhaps the term *superficial* can in a sense express the domain in which this happens: e.g., all at the surface, i.e., seeing as light strikes our eyes, hearing as sound waves reach our ears or feeling as some material touches our skin; or events at the surface, which is literally about our impressions and expressions at this surface. Moreover, we suggest that the theme “*the superficial that matters*” could be a way of pointing to these aspects. Is the superficial that we here suggest also unidentifiable as in the previous findings? Perhaps one could say that going too far with identification and interpretation would miss out precisely on the straightforwardness of aspects that are (and should remain) in the domain of the superficial.

It seemed as if this tone of voice, the eyes or the face offered them something decisive to overcome their difficulties and the momentum to go on—a “something” that made them dare to approach others and the world around them. Daring to be there, together with others, at the surfaces, offered a place in the world, taking up the offer and entering that place.

In everyday language, the term “superficial” has negative connotations and is an adjective that devalues something. It is not thorough, not thoughtful, not dealing with what is important, as opposed to “deep”. Bluntly, in this prevailing logic, “deep” implies something good, while “superficial” means something bad. In our view, this normative dichotomy between “superficial” and “deep” is misleading and may block our attention to important aspects of human living: e.g., sensing, touching, and moving at the surfaces; faces, voices, eyes and limbs. In everyday language, superficial and deep are used as metaphors. Our use of the term “superficial” is a return to a literal use and points to a domain of living that may be overseen by traditional metaphorical understandings where we want the deep and avoid the superficial.

This surface, it seemed, is described as both the “place” that we must be to live a good life and a “place” that makes living difficult. A man described his psychotic experiences as a kind of withdrawal from a terrifying surface:

I heard a murmur: a melody, maybe, a voice. I didn’t hear the words; they never go in. … The fear. … their eyes … aggressive tone … . And just bang! Straight in me. (T-2)

He continues by saying that this “fear” made him feel as if “a magnet was pulling him inwards”. At first as a positive and euphoric experience, but later it turned into a hurtful experience, as he said: “[It was] hell. No communication and one doesn’t get out”. From first being a euphoric escape from the reality (of surfaces), the psychotic world becomes even more terrifying than reality: “I fought tooth and nail to hold on to the world”.

This man also described the importance of his boss who had this gentle tone and he was asked to elaborate on this mildness, but surprisingly, he talked about cows:

Interviewer: When you speak of mildness, that one should be mild (…) what do you actually mean?

Man: It is something that …, something that always returns, a returning symbol or *it may be real*. I mean cows (…), it is important to pat cows on their velvet muzzle. (…) This is mildness. A cow’s eyes attract me somehow. (…) If I had been inside the bubble a lot now [I could have] said that one should plant cows here. (T-2)

It is in no way certain whether this statement points out the importance of the surface. Nevertheless, this man describes, as far as we see it, that when he is inside the (psychotic) bubble, what could get him out of the bubble and back to the world (i.e., the surface of the sensible) was something like the softness of the cow’s muzzle or the cow’s eyes that had always attracted him. At first, he suggested it was a symbol, but in the next breath, he questioned whether it could actually be real—the actual experience of mildness (of a cow) and not mildness as a symbol of something else. He is pointing out reality as the surface—the feeling and sensing of reality in its superficiality, not something any deeper and hidden. In a sense, he said he could come out of his psychosis through patting the muzzle of a cow and looking into its eyes. An event that would make it possible for him to let go of his psychotic constructions of another world and emerge from his bubble.

### *“She is so annoying!” (said with a big grin)*—nonsense that matters

A girl said that her friend had meant everything to her during the last year, and it was because of her that she had managed her difficulties. When asked what this was about, she said, “It’s incredibly much” and went on to say that “she is ‘weird’”, and then she described how her friend was just teasing her (her friend was also with her during the interview).

The girl: The humour. It’s so … It’s like I just want to hit her. But then it’s so funny at the same time. (…) For instance, like a couple of weeks ago. Then it was ‘douchebag’ or something like that, she went around saying.

Her friend: ‘Douchebag’.

The girl: ‘Douchebag’ over and over again all the time. (…) With lots of different voices (…). And she’s just so annoying (said with a big grin).

Friend: I’m doing it to annoy you.

The girl: That’s what’s so fun with her, but she can be annoying. Very annoying (said with a smile and playful glint in her eye: the girls made eye contact, revealing their shared enjoyment of the situation). (B-2)

Her answer to what her friend had meant to her, perhaps surprisingly, is neither about any practical help, nor about conversations related to her difficulties or advice. Instead, she goes straight into kidding and teasing and being “really weird” and irritating.

Humour, kidding and teasing—these are examples of the kinds of communication and interplay that are not related to sense-making or problem-solving. We thought that this could be pointed at using the theme *nonsense that matters*. It is as if the friend’s nonsensical foolishness offered a kind of invitation to be in vitalizing interplay outside any thinking about difficulties.

We tend to think of therapeutic conversations as conversations about “serious stuff”; going into the difficulties at hand in the life of the one seeking help. However, many participants spoke about talking about things totally aside from the difficulties, things seemingly unimportant that were important to them.

Eh … What we have done most often is that Mary (mental health practitioner) has picked me up after school and we’ve driven around for half an hour to an hour, so … then I feel we can talk about both personal things, as well as give attention to, like, things happening around us. In such a way that it’s not just about sitting down and talking seriously. That can be quite difficult. (B-3)

Is this about nonsense? We believe that perhaps an important aspect in what this girl said was how she appreciated—besides talking about herself and her difficulties—the possibility of also being together with the therapist without having to speak about “serious stuff”; simply being together, attentive to what was happening around them and talking about whatever they see around them without having to go into the difficult and without trying to sort something out or solve anything. In that sense, it could be seen as nonsense. The nonsenseness may show that there is a something going on between them beyond the problem-solving, sense-making enterprise of therapeutic conversations. A vitalizing nonsense that perhaps is about the two of them existing through each other’s presence.

## Discussion: invited to superficial existence?

This paper addresses the unidentifiable, the superficial and nonsense, perhaps pointing to something within human encounters that is embedded in the flesh and makes existing possible, but that simultaneously falls outside our attention when we give accounts of what is important in such encounters. We now propose that what escapes rationality and sense-making may belong to two domains, which we might call (1) human living as *superficial–corporeal* and (2) human living as *ethical–existential*. First, we reflect on the domains separately and then explore how they can be thought of together through the term (3) *invited to superficial existence.*

### Superficial–corporeal living

Let us start from a somewhat different point of departure. One of the authors was reading Proust’s *In Search of Lost Time* while doing this study and found that the quote below in a sense gives words to these sides of living that escape how we *think* about and *give meaning* to our living.

And then my thoughts, did not they form a similar sort of hiding-hole, in the depths of which I felt that I could bury myself and remain invisible even when I was looking at what went on outside? When I saw any external object, my consciousness that I was seeing it would remain between me and it, enclosing it in a slender, incorporeal outline which prevented me from ever coming directly in contact with the material form; for it would volatilise itself in some way before I could touch it, just as an incandescent body which is moved towards something wet never actually touches moisture, since it is always preceded, itself, by a zone of evaporation (Proust, 2002, p. 126).

In his novel, Proust explores memory, representations, (re)constructions of the world and others. We suggest that in this quote, Proust puts into words the same hermeneutic world loss that Gumbrecht (2004) pointed to. Proust writes from the position of the “hiding-hole” of his thoughts (hermeneutics), looking out at what goes on outside. As he approaches the outside reality through thoughts, this outside evaporates. If our relations to the world always and solely are our interpretations (thoughts) of it, then—for the “I think, therefore I am”—the real, the material–corporeal evaporates: a hermeneutic world loss.

Perhaps human living also could and should be seen as that which happens on “the other side”, the side that “evaporates” when approached by the glow of thought. Human living—and what matters to us—are perhaps just as much something that happen outside what we *think* of it. We move and are present within a “flow of materiality” (Ingold, 2010) prior to any naming, identifying or understanding.

Gumbrecht’s (2004) ideas of the hermeneutic world loss may shed light on our findings. When our interviewees speak of the mild tone of voice, the hostility in the voice, the feeling that they care, the appreciation that they can speak, and that they are listened to, that there is “something” between them, and so on, then perhaps this is about aspects of living and sensing that have their centre of gravity prior to people’s hermeneutic relationship to the world and others. Of course, one might object that the descriptions selected from the interviews also are in the domain of hermeneutics and interpretations (mild, friendly, hostile or caring). Still, as we see it, the descriptions highlight the corporeal–superficial and how they themselves exist and become in this domain even before any interpretation. One may also suggest that the tone of voice, e.g., should not be seen as the expression of some underlying friendliness; one might argue that the tone *is* the friendliness. The friendliness is neither an expression of an underlying (deep) quality nor an interpretation added. The friendliness coincides with the tone. It is all there at the surface.

Ingold (2017b) points out that there has been a remarkable revival of interest in surfaces in various disciplines in human and social sciences. This implies a shift from interest in the *knowledge* we have related to the world (epistemology) to the conditions of *being* (ontology). This shift entails that the “assumption that the true essence of things and persons is to be found deep inside of them” (Ingold, 2017b, p. 99) is challenged. Ingold (2017b) suggests that perhaps there is nothing underneath the surface. The surfaces may be the real sites and hence there is a danger that when we take a presumably deeper meaning to be what we are looking for, then what we seek becomes out of focus despite it being right there “under our very noses” (p. 100)—at the surface. We miss it because of the prevailing belief in “underlying meaning” according to which surfaces are something to be “excavated and cleared away” (p. 100).

When we give attention to surfaces, we sense their shifts and movements in “the continual birth of things” (Ingold, 2017b, p. 103). Ingold (2017b) writes that *social life* is lived in the relation between such surfaces, and goes on to suggest that a turn to the surfaces may entail “nothing less than a restoration; the restoration of social life itself” (p. 105).

Ingold (2017a) picks up the concepts of *minor gestures* and *major gestures* from Manning (2016). Minor gestures are those that make us “inhabitants” in a common world “before we ever find our feet in solid understanding” (p. 39). Minor gestures pull everyone and everything out of position; you are at risk, exposed—outside the security of understanding. These minor gestures are in danger of falling in the darkness, overshadowed by the *major gestures* of assertions, categorizations and explications.

This study started searching for the small things that often go unnoticed in accounts of what is important for people with mental health problems (Topor et al., 2018). It is possible that attention to social life as life at the surface and attention to minor gestures may help us to elucidate these small things, what they are about, where, when and how they occur. It is perhaps not a matter of deep understanding but one of superficial touch that interrupts and invites us into existence.

Some of our participants found it somewhat difficult to say what it is that was of importance to them; they stuttered, fumbled and ended up with blurry concepts like “something”, “quirky” or “a feeling that … ”. This could be seen as a matter of a lack of understanding of something that could be understood if one possessed the right concepts or theories. However, we speculate if we rather may see this as an indication of events in the domain of the superficial—materials, body, light, sound and movement—that have significance in their own right, and not as expressions of something deeper, and hence dependent on interpretation and sense-making.

### Ethical–existential living

Biesta (2014) suggests that human existence can be described as “to come into the world” and that this “coming into the world” only can be done “in the presence of others” (p. 143). Drawing on the ideas of Hannah Arendt, Biesta (2014) says that human actions are beginnings. In human action something new comes into the world. What we become through these beginnings is not in the hands of the originator but dependent on how our beginnings are picked up and responded to by others. “*We cannot act in isolation*. If I were to begin something but no one would respond, nothing would follow my initiative, as a result, my beginnings would not come into the world” (Biesta, 2014, p. 105). Biesta (2014) also refers to Emmanuel Levinas and points out that when encountering another human being, a call to take responsibility for the other always comes with it. Hence this event of existing in relation to others is always also ethical. It inevitably involves the question of how to take responsibility for the other.

Biesta (2016) also opposes the idea that human living is completely a matter of interpretation vis-à-vis the world, hence hermeneutics. Nevertheless, for Biesta, this does not entail a turn to the body, the surface, and materials in the sense that humans are immersed in and determined by the body and its material surroundings. According to Biesta (2016), human existence cannot be accounted for in terms of formation, as a course of vitality and growth in which humans develop, determined by the environments of materials, people and culture surrounding them. No, existing as subject—as an ethical event—is about the possibilities of humans to question and be questioned in this growth and their relations to their surroundings and others. Biesta (2018, italics added, no page number) says that “the human being *as subject* is not an acculturated organism, it is not a bio-neuro-social-cultural ‘entity’”.

His interest is in the ethical–existential dimension of being human, which he says can never be captured by any explanation or understanding even if it should include the whole range of “levels” from neurobiology to culture: “[T]he existential cuts through the bio-neuro-social-cultural level” (Biesta, 2018, no page number).

Existing as a subject is not about a person’s opinions, competences, roles, qualifications or abilities. Rather, existing as a subject is generated from “the outside” when someone addresses me and invites me to respond (Biesta, 2016). The subject comes into existence as responsibility towards the other and the outside world. The subject is an ethical *event* (rather than an ontological entity) in which I am “singled out”, as Biesta (2014) puts it, when addressed by another human being.

Where does this leave us given the exploration of this paper? Perhaps what we have noticed in the interviews and elaborated on could be about experiences of events of existence and ethics, events that interrupt the person as he/she is formed by “bio-neuro-social-cultural” determinants and that call for the subject—precisely me—to come forth in it all. The small things, the *nothings*, may be about experiences of how it is possible to exist in terms of “coming into the world in the presence of others” as Biesta puts it. This “nothing” or “something” that we suggest may be *unidentifiable*, events at the *surface* that strike me as a call into existence, and this *nonsensical* interplay with others the participants described; all this could be about events that made it possible to stay in the world. When tempted to give up the small things, the *nothings*, are invitations that called them to stay in the world.

Existence, then, seen as something new entering the world is not about something coming from any “higher” or “deeper” domain, but the exiting subject arrives in and from the midst of the encounters. Out of nothing so to speak. The subject arrives in a neuro-bio-social-cultural domain without being reducible to this domain. Existence is not about understanding and being understood, neither about forming and being formed. It is about the subject that arrives in and through the question “how can *I* take a next step in this?” Could it be that this event of the unique subject happens within the corporeal, sensible, moving life at the surface, not as a product of it, but as a split in it.

### Invited to superficial existence

Thus, is there a contradiction between the two domains that we have discussed: the superficial–corporeal and the ethical–existential? We suggest it is not. The domain of surfaces, materials and perception is also simultaneously the “site” of ethics and existence: “My boss had this mild tone in his voice”. From the perspective of the hermeneutic world-view, one may ask what lies behind or beneath this mild tone? What does it mean? What does it express? We may search for its underlying deeper meaning, but through such questions one becomes blind to the reality of the superficial. What happens at the surface deserves to be brought back into the light. Perhaps the tone *as* tone, something about the sound with its variations in pitch, volume or timbre is what makes the difference for the man, before any interpretation. Tone, not as something to understand, but tone as it arouses through the bodily movements of the other: making sound, carried through the air, reaching my ear and doing something to me. Tone as tone not as expression of something “behind” it.

What about ethics and existence? In this “vocal cords–voice–sound–ear–hearing happening”, the subject may arrive. The tone is sound, and at the same time a call, it is something that singles that man out and makes it possible for him “to subject” to the other and come into the world. The choice is imposed on him: “should I stay or should I walk away?” These are instances of ethical and existential incarnation of the subject. Called to exist prior to any sense-making. One might say that for the man, “the mild tone” of his boss interrupted him in his withdrawal from the reality of the surface and made it possible for him to choose to connect and respond again. A possibility opened up in the domain of the surface, through the singularity of sound, tone, light, warmth and contact.

To exist, etymologically, means to take a step forward, to come forth (Online Etymology Journal, 2018). Could we consider this in a literal way, not merely as a metaphor? Perhaps existing is coming forth literally in moving (Bøe et al., 2014). To live clearly means to constantly move; moving as the heart convulses, as the lungs exhale and inhale. Moving as the body takes steps, leaving places, entering new places, leaving the past and entering the future. So maybe existing is also literally about the way our body all the time moves into new spaces and into the future. It is in the midst of this movement that the existential question “should I stay or should I walk away” arises; in movement and at the same time as an interruption of movement. Existence also could—and should—be attended to in terms of movement and sensing within the domain of bodies, materials, sounds and light—the way the world strikes us, and in which we ourselves move into (exist) and make a difference. To exist is not to be understood, nor to understand, but to step forward—in the sense that my surface is made visible and both separable from and in contact with other surfaces. Invited into existence at the surfaces—invited to superficial existence.

## Concluding remarks

This paper started with the reading of some interviews with persons who described what had mattered much to them during times when life was difficult. We became interested in those sequences where they spoke of something important but that seemed difficult to put into words or seemed to fall outside the genres of hermeneutic coherent narratives of recovery. And we have explored what might be concealed by the hermeneutical world-view.

Continuing these speculations, we could ask what implications this could have for practice and research in mental health. If research in mental health rules out what cannot be articulated in terms of knowledge, then perhaps there is a danger that the field is not only enriched by research, but also in a sense impoverished. As we have suggested in this paper, there might be “something” that makes it possible to “stay in the world” and makes existence possible that is found outside the knowledge that any research can produce. Mental health is a complex domain and include a wide range of phenomena, from what must be considered neurological diseases on one side and relational and societal related difficulties on the other side. We are reluctant to make any definite claims about implications for practice. Further it should be made clear that we don’t suggest any new paradigm that should replace other rationalities. But we try to point out something important that may be missed in prevailing approaches in research and practice.

Nevertheless, we could question any one-sided reliance on method-based approaches based on quantitative research built on an instrumental logic of intervention. Furthermore, we could also question the growing focus on person-oriented and recovery-supporting approaches based on qualitative research. This research and associated approaches are also based on the idea of generating knowledge of recovery processes based on identification of what works, in this case, from research exploring the person’s own narratives. Perhaps there is something that matters much that cannot be captured by experimental designs (producing evidence for methods) or by qualitative designs (making sense of people’s lives). A *something else* beyond the framework of identification and knowledge should be allowed to remain *nothing*, but *a nothing that matters*.

## Notes

1. It should be noted that such reflections on recovery are also articulated in the recovery literature, as in Davidson, Tondora, and Ridgway (2010).

2. We understand the term “ordinary therapeutic rationality” in line with the instrumental and hermeneutic rationalities described above.

3. We indicate what part of the empirical material the various quotes and examples are drawn from. The codes B, T and L indicate the authors, respectively, followed by interview number.

4. All names in quotes from the interviews are pseudonyms.

5. This boy’s description of the practitioner is elaborated on by Bertelsen and Bøe (2016).

## References

[CIT0001] Bertelsen B., & Bøe T. D. (2016). ‘He is quirky; He is the world’s greatest psychologist’: On the community of those who have nothing in common. *Australian and New Zealand Journal of Family Therapy*, 37(3), 367–11.

[CIT0002] Biesta G. (2014). *The beautiful risk of education*. London: Paradigm Publishers.

[CIT0003] Biesta G. (2016). The rediscovery of teaching: On robot vacuum cleaners, non-egological education and the limits of the hermeneutical world view. *Educational Philosophy and Theory*, 48(4), 374–392.

[CIT0004] Biesta G. (2018). The need for ‘Pedagogikk’ – And why no other word will do. Lecture February 13th, 2018, at Bergem-seminar, NLA Bergen, Norway.

[CIT0005] Biong S., & Ravndal E. (2007). Young men’s experiences of living with substance abuse and suicidal behaviour: Between death as an escape from pain and the hope of a life. *International Journal of Qualitative Studies on Health and Well-being*, 2(4), 246–259.

[CIT0006] Bøe T. D., Kristoffersen K., Lidbom P. A., Lindvig G. R., Seikkula J., Ulland D., & Zachariassen K. (2013). Change is an ongoing ethical event: Levinas, Bakhtin and the dialogical dynamics of becoming. *Australian & New Zealand Journal of Family Therapy*, 34(1), 18–31.

[CIT0007] Bøe T. D., Kristoffersen K., Lidbom P. A., Lindvig G. R., Seikkula J., Ulland D., & Zachariassen K. (2014). “She offered me a place and a future”: Change is an event of becoming through movement in ethical time and space. *Contemporary Family Therapy*, 36(4), 474–484.

[CIT0008] Bøe T. D., Kristoffersen K., Lidbom P. A., Lindvig G. R., Seikkula J., Ulland D., & Zachariassen K. (2015). ‘Through speaking, he finds himself … a bit’: Dialogues open for moving and living through inviting attentiveness, expressive vitality and new meaning. *Australian and New Zealand Journal of Family Therapy*, 36(1), 167–187.

[CIT0009] Davidson L., Tondora J., & Ridgway P. (2010). Life is not an “outcome”: Reflections on recovery as an outcome and as a process. *American Journal of Psychiatric Rehabilitation*, 13(1), 1–8.

[CIT0010] Freeman M. (2013). Returning the other to the story of the self. Paper presented at the Levinas Lectures, Lesley University Retrieved from http://www.youtube.com/watch?v=V-Y19iGu-nk

[CIT0011] Gumbrecht H. U. (2004). *Production of presence: What meaning cannot convey*. Stanford, CA: Stanford University Press.

[CIT0012] Herrestad H., & Biong S. (2010). Relational hopes: A study of the lived experience of hope in some patients hospitalized for intentional self-harm. *International Journal of Qualitative Studies on Health and Well-being*, 5(1), 1–9. 10.3402/qhw.v5i1.4651 PMC2879868 20640026

[CIT0013] Ingold T. (2007). Materials against materiality. *Archaeological Dialogues*, 14(1), 1–16.

[CIT0014] Ingold T. (2010). Bringing things back to life: Creative entanglements in a world of materials. Retrieved from http://eprints.ncrm.ac.uk/1306/1/0510_creative_entanglements.pdf

[CIT0015] Ingold T. (2017a). *Anthropology and/as education*. London: Routledge.

[CIT0016] Ingold T. (2017b). Surface visions. *Theory, Culture & Society*, 34(7–8), 99–108.

[CIT0017] Knausgaard K. O. (2018). *The end: My struggle. Book 6*. London: Vintage Publishing.

[CIT0018] Larner G. (2008). Exploring Levinas: The ethical self in family therapy. *Journal of Family Therapy*, 30, 351–361.

[CIT0019] Larsen I. B. (2009). *“Det sitter i veggene”. Materialitet og mennesker i distriktspsykiatriske sentra* [It is embedded in the walls. Materiality and people in district psychiatric centres.] Bergen: The University of Bergen.

[CIT0020] Manning E. (2016). *The minor gesture*. Durham: Duke University Press.

[CIT0021] Norcross J. C., & Wampold B. E. (2011). Evidence-based therapy relationships: Research conclusions and clinical practices. *Psychotherapy (Chic)*, 48(1), 98–102. 21401280 10.1037/a0022161

[CIT0022] O’Mahony S. (2017). Medicine and the McNamara fallacy. *The Journal of the Royal College of Physicians of Edinburgh*, 47(3), 281–287. 29465108 10.4997/JRCPE.2017.315

[CIT0023] Online Etymology Dictionary (2018). Existence (n.). Retrieved from https://www.etymonline.com/word/existence

[CIT0024] Proust M. (2002). *Swann’s way*. North Chelmsford, MA: Courier Corporation.

[CIT0025] Rober P. (2005). The therapist’s self in dialogical family therapy: Some ideas about not-knowing and the therapist’s inner conversation. *Family Process*, 44(4), 477–495. 16433290 10.1111/j.1545-5300.2005.00073.x

[CIT0026] Sælør K. T., Ness O., Holgersen H., & Davidson L. (2014). Hope and recovery: A scoping review. *Advances in Dual Diagnosis*, 7(2), 63–72.

[CIT0027] Schon U.-K., Denhov A., & Topor A. (2009). Social relationships as a decisive factor in recovering from severe mental illness. *International Journal of Social Psychiatry*, 55(4), 336–347. 19553363 10.1177/0020764008093686

[CIT0028] Seikkula J. (2002). Open dialogues with good and poor outcome for psychotic crises: Examples from families with violence. *Journal of Marital and Family Therapy*, 28(3), 263–274. 12197148 10.1111/j.1752-0606.2002.tb01183.x

[CIT0029] Seikkula J. (2011). Dialogue is the change: Understanding psychotherapy as a semiotic process of Bakhtin, Voloshinov and Vygotsky. *Human Systems*, 22(2), 521–533.

[CIT0030] Seikkula J., & Trimble D. (2005). Healing elements of therapeutic conversation: Dialogue as an embodiment of love. *Family Process*, 44(4), 461–475. 16433289 10.1111/j.1545-5300.2005.00072.x

[CIT0031] Skatvedt A. (2017). The importance of “empty gestures” in recovery: Being human together. *Symbolic Interaction*, 40(3), 396–413.

[CIT0032] Skatvedt A., & Scheffels J. (2012). Virksom uvirksomhet? Pauser som arenaer for følelsesmessig berøring og biografisk bevegelse. [Working inactivity. Pauses as arenas for emotional touch and biographical movement.]. *Sosiologi i Dag*, 42(1), 37–56.

[CIT0033] Skatvedt A., & Schou K. C. (2008). The beautiful in the commonplace. *European Journal of Cultural Studies*, 11(1), 83–100.

[CIT0034] Sullivan P. (2012). *Qualitative data analysis using a dialogical approach*. London: Sage Publications.

[CIT0035] Topor A., Bøe T. D., & Larsen I. B. (2018). Small things, micro-affirmations and helpful professionals everyday recovery-orientated practices according to persons with mental health problems. *Community Mental Health Journal*, 54(8), 1212–1220. 29423684 10.1007/s10597-018-0245-9 PMC6208994

[CIT0036] Topor A., & Denhov A. (2015). Going beyond: Users’ experiences of helping professionals. *Psychosis*, 7(3), 228–236.

[CIT0037] Wampold B. E., & Imel Z. E. (2015). *The great psychotherapy debate: The evidence for what makes psychotherapy work*. London: Routledge.

[CIT0038] Yankelovich D. (1972). *Corporate priorities: A continuing study of the new demands on business*. Stanford, CT: Yankelovich Inc.

